# NiTi Shape Memory Clamps with Modified Surface for Bone Fracture Treatment

**DOI:** 10.3390/ma16165575

**Published:** 2023-08-11

**Authors:** Tomasz Goryczka, Tomasz Szponder, Karolina Dudek, Tadeusz Wierzchoń, Jarosław Paluch, Krzysztof Jasik, Ryszard Wiaderkiewicz

**Affiliations:** 1 Institute of Materials Engineering, University of Silesia in Katowice, 75 Pułku Piechoty 1A, 41-500 Chorzów, Poland; karolina.dudek@icimb.lukasiewicz.gov.pl; 2 Department and Clinic of Animal Surgery, Veterinary Medicine Faculty, University of Life Sciences, Akademicka 13, 20-950 Lublin, Poland; tomasz.szponder@up.lublin.pl; 3 Łukasiewcz Research Network, Institute of Ceramics and Building Materials, Cementowa 8, 31-983 Chorzów, Poland; 4 Faculty of Materials Science and Engineering, Warsaw University of Technology, Wołoska 141, 02-507 Warszawa, Poland; tadeusz.wierzchon@pw.edu.pl; 5 Department and Clinic of Laryngology, Medical University of Silesia, Francuska 20-24, 40-027 Katowice, Poland; j.paluch@sum.edu.pl; 6 Department of Pathology, Faculty of Pharmaceutical Sciences in Sosnowiec, Medical University of Silesia, Ostrogórska 30, 41-200 Sosnowiec, Poland; kjasik@sum.edu.pl; 7 Department of Histology and Embryology, Medical University of Silesia, Medyków 18, 40-752 Katowice, Poland; wiader@sum.edu.pl

**Keywords:** NiTi shape memory alloy, shape memory clamps, in vivo, bone-fracture joining

## Abstract

Using NiTi alloys with shape memory for long-term medical implants requires modification of their surface due to the possible occurrence of corrosion. Hence, the surface of the staples used to join fractured bone within the craniofacial region was modified by applying a titanium oxy-nitrogen layer and a hydroxyapatite coating. Surface-modified clamps were tested in vivo using New Zealand white rabbits. After determining the mechanical characteristics of the bone and considering the initial state and surface modification, the diameter of the wire (used to make the clamps with the appropriate compression force) was selected. Implantation was performed on two groups of rabbits: experimental and control. In the experimental group, an intentionally induced bone fracture was treated in one tibia. On the second tibia, two additional clamps were applied to increase the possibility of a negative impact of the NiTi alloy on a living organism. After 6 weeks of application, a proper joining of the broken bone fragments was stated. Whereas after twelve weeks, no negative impact of the clamp material on a living organism, i.e., a rabbit, was found. Hence, the clamp with the modified surface can connect bone fragments in humans as well as small and medium-sized animals, with an extended range of use up to 12 weeks.

## 1. Introduction

The number of practical applications of NiTi alloys in medicine, veterinary medicine, and medical instruments is growing yearly [1,2,3,4]. It concerns alloys with a chemical composition close to equiatomic with a slight predominance of nickel content—a few tenths of a percent [5]. Such a chemical composition guarantees the occurrence of a reversible martensitic transformation below room temperature. Thus, the shape memory effects can be used in the temperature range of the human body [6]. In medical applications, the one-way shape memory effect and superelasticity are the most often used phenomena [7]. As a driving force to change their shape, the implants use the body temperature of the living organism. On the other hand, blocking the possibility of returning to the initially programmed shape while increasing the temperature translates into the possibility of force recovery. These phenomena are used in practical medical applications such as popular stents, orthodontic wires, clamps for joining bone fragments, and distraction rings [1,3,5,7].

However, when the NiTi element is left in the living organism for an extended period, it may interact with the tissues and body fluids. This effect intensifies as the time of application increases. In extreme cases, this condition can lead to corrosion. As a result, the released nickel ions may negatively affect the living organism by causing local toxicity, inflammation, or allergies in hypersensitive patients [8,9,10]. A way to prevent material corrosion can be the application of protective layers/coatings made from biotolerant materials. So far, the coatings have been created in the form of a layer based on titanium oxides [11], titanium nitrides [12], titanium oxy-nitrides [13], diamond-like layers [14], hydroxyapatite [15], and biocompatible polymers (i.e., polylactide, chitosan) [16]. In the case of titanium oxide, nitride, and oxy-nitride layers, it is necessary to introduce the technology, which requires increasing the processing temperature. Methods such as powder immersion reaction-assisted coating [12], conducting processes in a reactive atmosphere [17], plasma ion implantation [18], or laser surface treatment carried out in an appropriate atmosphere [11,19] are known and used for NiTi surface modification. However, those techniques required high-temperature heat treatment, even at 900–1000 °C. This state affects the behavior of shape memory effects and the temperature range of their occurrence. Moreover, it leads to the formation of equilibrium phases enriched in nickel in the near-surface areas. Such sub-layers lead to a decrease in mechanical properties and layer delamination. In consequence, it deteriorates the mechanical properties of the composite.

Although carried out at relatively low temperatures, the deposition of diamond-like layers by ion implantation leads to similar effects. The stresses accumulated in the zone near the surface also contribute to layer cracking. On the other hand, polymer layers do not require elevated temperatures during their deposition. However, their adhesion in the case of cyclical repetition of shape memory phenomena may be insufficient. Similarly, the deposition of the hydroxyapatite layers does not require an increase in temperature and is carried out at room temperature, for example, by electrophoretic deposition. In this case, the increase in adhesion is obtained by short-term annealing at 700–800 °C, with a low influence on the martensitic transformation behavior [15].

The glow discharge technique is promising for depositing titanium oxide, nitride, and titanium oxy-nitride layers. Conducted at relatively low temperatures, it makes it possible to create titanium-based layers that adhere very well to the substrate. In addition, these layers are characterized by excellent mechanical properties and increased corrosion resistance concerning the unprotected NiTi matrix.

These properties can be used to increase surface protection by creating multifunctional layers consisting, for example, of a layer of titanium oxy-nitride with a layer of hydroxyapatite formed on it. The titanium oxy-nitride provides good mechanical properties and corrosion resistance, and the hydroxyapatite coating increases the biocompatibility of the entire composite. Thus, in the case of our research, an attempt was made to use a NiTi alloy with a modified, multifunctional surface for clamps used primarily in maxillofacial surgery to join bone fragments. Hence, the research was guided by two goals. The first was to determine the impact of the protective layers on the implant’s environment. Ultimately, clamps with a modified surface would be dedicated to joining bone fragments in maxillofacial surgery. It mainly concerns cases where the treatment would be carried out for a long time, requiring application in the body for longer than the standard six weeks. In this case, it concerns the migration of nickel ions, which is unfavorable for living organisms and proves the protective effect of the applied layers/coatings. Such an approach required conducting in vivo studies.

The first goal emerged as the possibility of additional testing of the joining bone fragments with shape memory clamps in the case of hind limb fractures in small animals. Domestic rabbits were selected to achieve both goals. They are characterized by high bone fragility, which in some cases limits the use of surgical plates or external stabilizers [20]. Compared to traditional methods used for domestic animals, the use of modified shape memory clamps gives the possibility of selecting the appropriate pressure force adapted to the bone characteristics already at the stage of clamp design.

## 2. Materials and Methods

### 2.1. Clamp Production and Their Characterization

**Figure 1:**
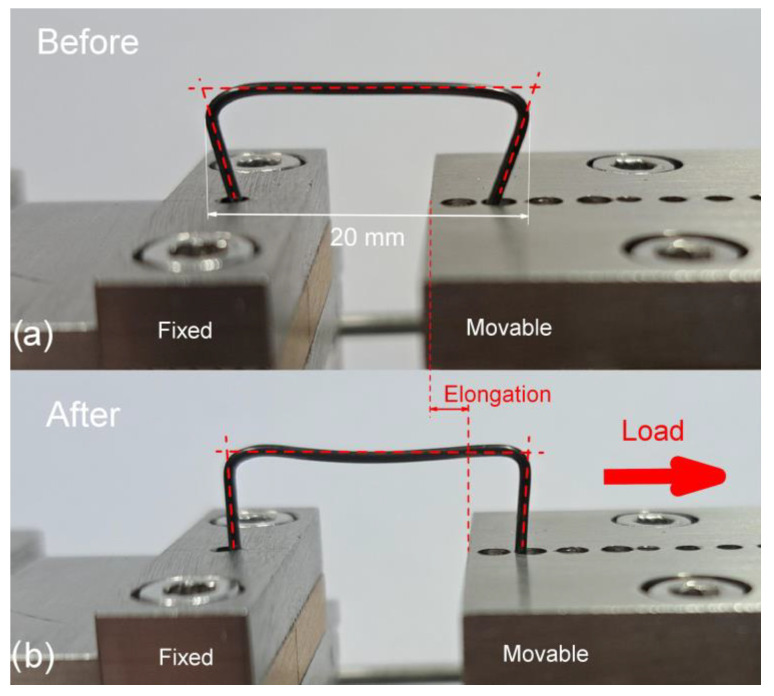
Images of an example clamp with the initial shape (before) (**a**) and deformed to the “U” one (after) (**b**).

Commercial NiTi wires with a diameter from 0.8 mm to 1.7 mm were used for clamp production. They were formed in the initial shape—with clenched arms, shown in Figure 1a. Before implantation, the clamps were “U”-shaped (Figure 1b). The long span measured from 20 to 25 mm, while the arms of the clamp were from 5 mm to 10 mm.

The surface of the clamps was modified in two stages. First, a titanium oxy-nitride layer was formed using a low-temperature glow discharge treatment. The applied process led to the formation of a protective layer consisting of two sub-layers. The nanocrystalline titanium nitride was created first as a sub-layer directly on the substrate, then as amorphous titanium oxide. The total thickness of both sub-layers was about 25 nanometers. Technological parameters can be found in [13]. The titanium oxy-nitride was covered in the second stage with a hydroxyapatite layer (HAp). The HAp layer was applied by electrophoresis. Using a voltage of 30 V to 80 V and a deposition time of about 30 s to 180 s provided the conditions for creating coverage with a thickness of about tens of micrometers. The final stage of preparation of the HAp coating was sintering at 800 °C for 2 h [15]. The characteristics of the clamps were carried out in the initial state and in the final state—after applying the titanium oxy-nitride and hydroxyapatite layers (final state).

For implantation purposes, ten sets of clamps covered with a layer of titanium oxy-nitride and hydroxyapatite were prepared for lengths of 20 mm and 25 mm, respectively. The series included clamps with arm lengths ranging from 5 mm to 10 mm (every 1 mm). During the implantation procedure, the surgeon could choose the appropriate size of the clamp according to the dimensions of the rabbit bone.

The mechanical behavior of the clamps and bones was tested on a Mikrotest device (Deben UK Limited, Suffolk, UK) equipped with a 200 N load cell under the conditions specified in the ASTM F2516 standard. The tests made it possible to determine the maximum force needed to form the “U”-shape, the value of the force recovered during the one-way shape memory effect, and the force of elastic deformations of the bones. The way of the clamp attachment and measurements are shown in Figure 1.

The martensitic transformation courses and the one-way memory effect behaviors at each stage of their preparation were determined based on the measured thermograms. Measurements were performed using a Mettler Toledo DSC 1 (Greifensee, Switzerland) calorimeter. Samples weighing 30 to 50 mg were heated/cooled at 10 °C per minute in a protective argon atmosphere in the temperature range from −120 °C to 160 °C.

### 2.2. Clamp Implantation and Biological Material Preparation

The studies were carried out on six-month-old New Zealand White male rabbits with an average body weight of 3.4 kg (3.1 to 4.2 kg) at the Department and Clinic of Animal Surgery, Faculty of Veterinary Medicine, University of Life Sciences in Lublin, with the consent of the 2nd Local Ethical Committee for animal experiments (13/2014). Animals were maintained following the International Council for Laboratory Animal Sciences Guidelines for the Care and Use of Laboratory Animals.

The rabbits were divided into two groups: an experimental group (10 individuals) and a control group (2 individuals). Individuals in the experimental group were subjected to clamp implantation, whereas individuals in the control group were used as comparative materials. After the end of the experiment, all animals were euthanized.

Anesthesia was premedicated with medetomidine (0.5 mg/kg b.w. i.m., Domitor, Orion Pharma, Finland) and butorphanol (0.2 mg/kg b.w. i.m., Butomidor, Richter Pharma, Austria). Ketamine (30–50 mg/kg b.w., i.v.; Bioketan, Biowet, Poland) was used for general anesthesia. In the first hind limb, medial access to the tibia was performed under sterile conditions, followed by mid-length osteotomies. Bone fragments were stabilized using Kirshner intramedullary nails (Mikromed, Dąbrowa Górnicza, Poland) and two clamps implanted near the fracture line. The correctness of the anastomosis was confirmed by a radiological examination (Dongmun DIG 360, 2 kV, 50 Ms). In the second hind limb, two clamps were implanted into the tibia after medial access to enhance the possible unwanted effect of nickel on the biological environment. The postoperative wound was sutured in a standard manner. After surgery, rabbits were given Torbugesic (Pfizer Polska, Warsaw, Poland) as an analgesic and Sulfatrim (Scanvet Polska, Warsaw, Poland) to avoid infection. Control radiologic examinations were performed after 6 and 12 weeks. After 12 weeks, the animals were euthanized (150 mg/kg i.v. Morbital, Biowet Puławy, Puławy, Poland). Radiological examinations were performed, and material for histological examinations was collected. Macroscopic observations and evaluation of the tissues at the implantation site were performed during the material collection.

Bone samples were studied with a high-resolution X-ray scanner (v|tome|xs, GE Sensing & Inspection Technologies, Phoenix|X-ray, Wunstorf, Germany) operated at 200 kV and 180 μA. The samples were adjusted to a holder. The 2000 projections were registered with optimal contrast and a resolution of 10 μm. Projection acquisition was performed using specialized software (GE Sensing & Inspection Technologies, Wunstorf, Germany). Reconstruction and visualization were performed in 8-bit grayscale with VGStudioMAX 2.1 software (Volume Graphics, GmbH., Heidelberg, Germany). Computed microtomography made it possible to visualize the bone microstructure and the implanted clamps.

### 2.3. Histological Studies

Approximately 2 to 3 cm long tibia fragments were used for histological examination. These fragments were fixed in 4% neutral-buffered formalin (phosphate buffer) for five days and then decalcified for 24 h in 14% HCl (Shandon TBD-1 Rapid Decalcifier, Thermo Scientific, Waltham, MA, USA). After extensive washing for 24 h in tap water, the tissue fragments were processed by passing through graded alcohol solutions, xylene, and finally embedded in paraffin blocks. Slices of 7 μm thickness were placed on silane-coated slides, deparaffinized, rehydrated, and stained with hematoxylin (15 min) and eosin (10 min). The photographic documentation was performed with a DP-21 camera (OLYMPUS, Tokyo, Japan) coupled with an Eclipse E400 (NIKON INSTRUMENTS, Inc., Tokyo, Japan) optical microscope. In order to test the presence of nickel, which could be the result of migration into bone tissues, the biological material was collected after removing the clamps. Appropriate samples were cut with a surgical electric knife. For the sample preparation process to not affect the tested chemical composition, it was decided to omit the chemical surface treatment. Samples were only dried in the atmosphere with the silica gel desiccant at 30 °C for three days. Next, the specimens were carbon coated in a high vacuum coater (Jeol Ltd., Tokyo, Japan) and examined by scanning electron microscopy (JSM-7100 F TTL LV, Jeol Ltd., Tokyo, Japan) equipped with a NORAN Vantage energy dispersive spectrometer (EDS). The microscope was operating at 15 kV.

## 3. Results and Discussion

### 3.1. Mechanical Characteristics of the Rabbit Tibia

**Figure 2:**
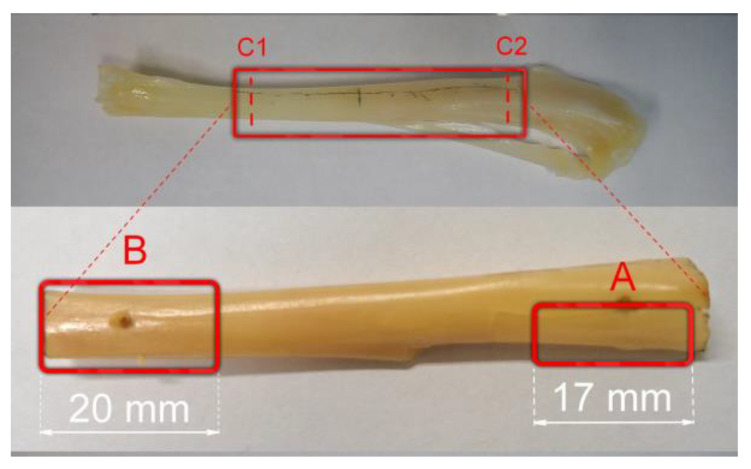
Photo of the tibia with marked fragments used for compression testing: compact bone “A” and the bone with yellow bone marrow “B”.

It is known that the rabbit is one of the most mobile pets. The weight of its skeleton is only about 7 to 8 percent of the total weight of the rabbit. The hind limbs of rabbits are characterized by unusual musculature and carry about 70% of the entire body weight. However, the bone structure is exceptionally delicate [4]. Therefore, in the first research stage, the spatial geometry of the internal bone and the mechanical characteristics of the implanted bone were determined (Figure 2).

**Figure 3:**
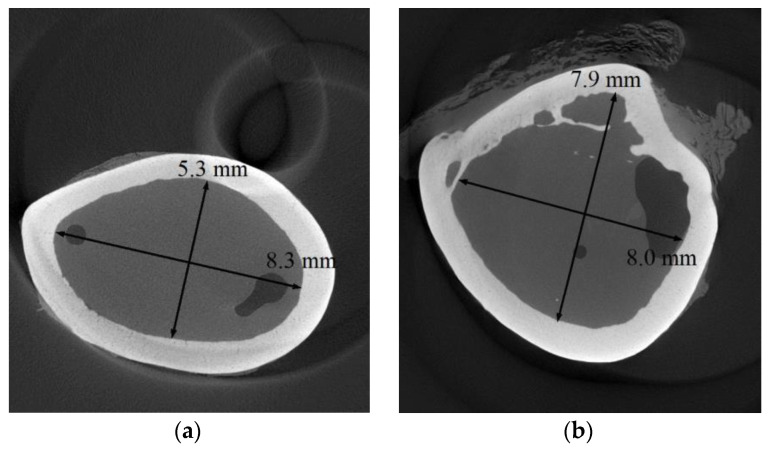
CT micrographs made on sections C1 (**a**) and C2 (**b**) (marked in Figure 2).

The geometry of the space where the clamps had to be implanted was determined based on microphotographic examinations. Example images observed in the sections marked C1 and C2 (Figure 2) are shown in Figure 3. The sections of the space filled with bone marrow were elliptical, in which the semi-major axis was about 8 mm and was characterized by an almost constant value along the entire length of the examined bone. In contrast, the semi-minor axis length varied over the length of the examined bone fragment from 5 to 8 mm. Also, based on tomographic images, the average compact bone thickness was determined, which was 0.99 ± 0.11 mm.

The results were the basis for designing the implant clamp and joining broken bone fragments. First, the length of the arms and the angle of their curvature were determined. It was assumed that for practical implantation, the arms of the clamp should not be longer than 8 mm with a bend angle of about 60°.

The fragments marked in Figure 2 as areas “A” and “B” were taken to characterize the mechanical properties. An approximately 17 mm section of compact bone with a width of 8 mm was taken from area “A”, while from area “B”, the bone with yellow bone marrow was approximately 20 mm long. In addition, the “B” area has been weakened by drilling a hole in the same way that holes for mounting a clamp are made during the operation.

**Figure 4:**
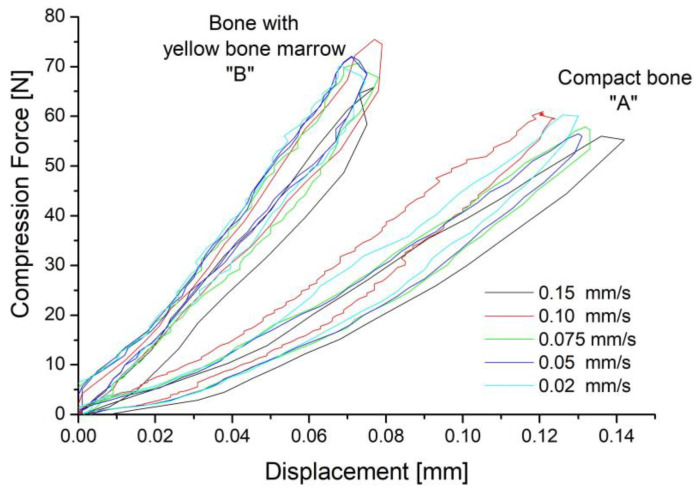
Result of the static compression test carried out (for various compression rates) for the compact bone “A” and the bone with yellow bone marrow “B”.

During the clamp’s operation, it presses the broken fragments and affects the bone in compression mode. Hence, the range of bone deformations was tested in the static compression test. The test was carried out cyclically at a different rate of force application. Five loading/unloading cycles were performed for each speed. The example results are summarized in Figure 4.

The compact bone “A” fragment was more susceptible to elastic deformations than the bone fragment containing the yellow bone marrow “B”. It was deformed by almost 0.8% under a force of about 60 N. A complete bone containing bone marrow shows a different character. Due to the space filling with bone marrow, a smaller elastic deformation was obtained with a force of about 75 N. The results show that the bone is susceptible to cyclic elastic deformations. However, the force’s value was the main factor in determining the appropriate wire selection, which should not exceed 60 N. Notably, despite using a different strain rate, the mechanical characteristics of elastic strains were similar. Consequently, the deformation rate does not play a significant role in the examined range of elastic deformations. This is essential when the clamp implantation process uses the one-way shape memory effect or superelastic behavior. In the case of a one-way shape memory effect, it will contract at a much slower rate than in the case of superelasticity.

### 3.2. Characteristics of the Wire Used for Clamp Production

As shown in previous works, the value of the force that can be recovered during the one-way shape memory effect depends on the diameter of the wire [7]. Therefore, following the parameters obtained from the mechanical characteristics of the bones, NiTi alloy wires with a diameter in the range of 0.8 mm to 1.7 mm were used to form the clamps. In addition to the wire diameter, the chemical composition of the alloy was an important aspect—it determined the temperature range of the reversible martensitic transformation. It is a known fact that an increase in the nickel content by 0.1% at the cost of titanium causes a shift of the characteristic martensitic transformation temperatures by about 20 °C towards lower temperatures [5]. Therefore, in order to ensure the occurrence of shape memory phenomena in the appropriate temperature range, adapted to the temperature of the human and rabbit bodies, it was necessary to use a NiTi alloy with a nickel content ranging from 50.3 at.% to about 50.8 at.% for the tests. This chemical composition ensures the one-way shape memory effect below ambient temperature. Consequently, it guarantees the presence of the parent phase at room temperature, a prerequisite for superelasticity.

The determined chemical compositions for individual wires are shown in Table 1. All alloys revealed a predominance of nickel content. Thus, the condition ensuring the occurrence of shape memory phenomena was met in the range adapted to the temperature range of the human and rabbit body temperatures.

**Figure 5:**
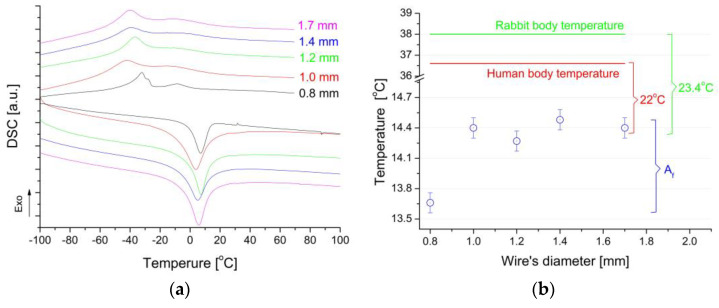
Thermograms measured for clamps in the initial state (**a**) and comparison of the difference between the temperature A_f_ and the temperatures of the rabbit and the human bodies (**b**).

From the point of view of the practical application of the NiTi alloy for medical implants, the temperature at the end of the reverse martensitic transformation (A_f_) must be relatively low concerning the body temperature of a living organism. This condition forces the entire transformation to occur below room temperature. Its consequence is completing the reversible martensitic transformation in the temperature range of about −60 °C to −80 °C. The course and range of the reversible martensitic transformation occurrence for wires were determined based on the measured thermograms (Figure 5a). The results proved that in all wires, there was a reversible martensitic transformation, which met the primary condition for the occurrence of shape memory phenomena. This transformation occurs in two stages during cooling, while only one stage was observed during heating. This condition often appears in wires where the manufacturing technology increases the density of line defects. Their presence causes an increase in the local stress state, which, among other things, is the reason for the appearance of a two-stage transformation through the R-phase. Generally, the transformation occurred between room temperature and −60 °C. This range guarantees the possibility of the clamps being shaped using liquid nitrogen and returning to their original shape by heating over the Af temperature with heat from the living organism.

A detailed analysis of the temperature range was made by calculating the difference between the A_f_ temperature and the human and rabbit body temperatures. The results are summarized in Figure 5b. The A_f_ temperatures of wires with a diameter from 1.0 to 1.7 mm were in the range of 14°. Compared to the temperatures of the human and rabbit bodies, the differences were 22° and 23°, respectively. This is a sufficient temperature reserve to use selected wires for clamp production. At this point, it should also be noted that not only the characteristic temperatures were the main factor in selecting the appropriate wire. The most important parameter was the force value that can be recovered from the deformed clamps when they returned to their initially programmed shape. It must be adjusted to the elastic range of bone deformation.

### 3.3. Determination of the Clamp’s Acting Force

**Figure 6:**
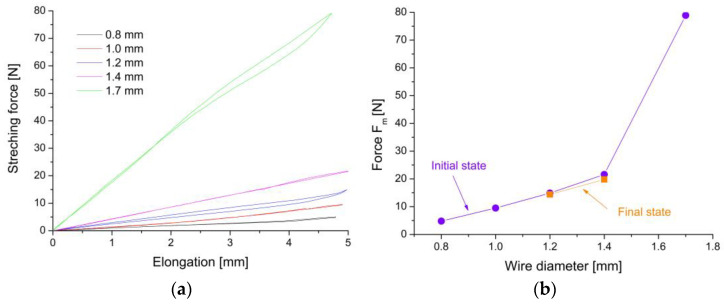
Stretching force versus elongation measured for clamps made of wire with a diameter from 0.8 mm to 1.7 mm (**a**) and the maximum force leading to the “U” shape (**b**).

The force needed to deform the clamp to the “U” shape was determined from the tensile test. The measurements were performed at room temperature following the scheme shown in Figure 1. The clamp was stretched until it reached the final “U” shape (Figure 1b). The results of the determined force depending on the elongation are presented in Figure 6a. The obtained characteristics show that for clamps made of wires with a diameter of 1.2 mm to 1.7 mm, the acting force induced superelastic behavior with a characteristic close loop. According to the increase in the wire’s diameter, the value of the force also increased. The maximum force values for individual clamps are summarized in Figure 6b. For the thinner wire (0.8 mm), the shape was received with 6 N only. The increase in the wire’s diameter caused an increase in the force value. Consequently, for a clamp with a diameter of 1.7 mm, the force was already about 80 N. It reached a value comparable to the range of determined elastic deformations of the rabbit’s bones. For clamps made of wire with a diameter of 1.0 mm, it was about 10 N; for 1.2 mm and 1.4 mm, it was 15 N and 22 N, respectively.

**Figure 7:**
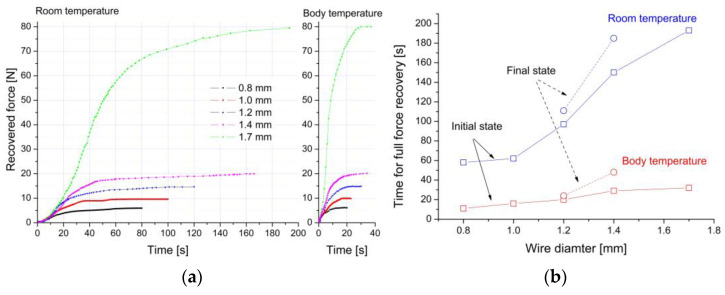
Recovered force versus the time needed for shape recovery, measured at room and living organism temperatures for the initial state of wires (**a**) and the time needed to full recovery force versus wire diameter (**b**).

An essential element in the characteristics and selection of the diameter of the wires for clamp production is also the time after which the “U” shaped clamp will return to its original shape. Such a characteristic was determined by measuring the time needed for shape recovery. First, the clamp was “U” shaped at liquid nitrogen. Then, it was freely heated to room temperature. In this case, the one-way shape memory effect was used. Additionally, the temperature conditions of the human and rabbit bodies were simulated by warming the clamps with hot air to 37 °C. The measurements were repeated. The determined characteristics are summarized in Figure 7a. On this basis, the time after which the clamps start working with their maximum force was determined and is shown in Figure 7b.

The course of the characteristics measured at room temperature show that, regardless of the diameter of the wire, the acting force begins to work after about a few seconds. In contrast, raising the temperature to the temperature of a living organism causes an immediate increase in strength. Depending on the diameter, the time required for force recovery increased with the increasing diameter of the wire. It ranged from 60 to 200 s for 0.8 mm and 1.7 mm, respectively. This effect results from differences in heat capacity, which increases with the diameter of the wire. In the case of temperature conditions prevailing in a living organism, this time was 30 s at maximum for the clamp with the highest heat capacity (1.7 mm). For clamps made of 1.2 mm and 1.4 mm wires, this time was shortened to about 20 and 30 s, respectively. A relatively short time leading to the maximum clamping force enables efficient and quick immobilization of bone fragments, protecting them against displacement.

### 3.4. Characteristics of Clamps after Surface Modification

**Figure 8:**
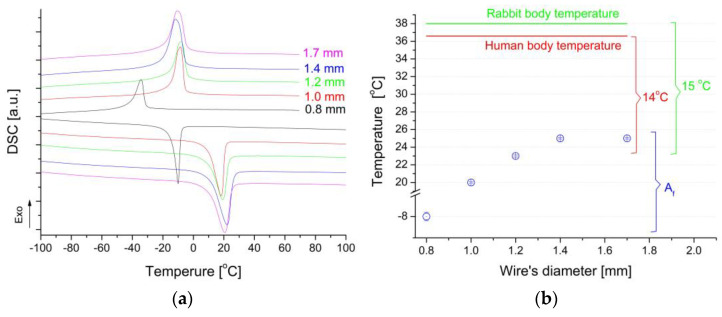
Thermograms measured for clamps after surface modification (final state) (**a**) and comparison of the difference between the temperature A_f_, the temperatures of the rabbit, and the human body (**b**).

The main objective of the research was to modify the surface with layers/coatings aiming to create barrier against the potential migration of nickel ions released. Therefore, the surface of the clamps was first covered with a layer of titanium oxy-nitride. Then a hydroxyapatite coating was applied. Following the Ni-Ti phase equilibrium system, the parent phase is metastable at room temperature. Hence, each increase in temperature causes the formation of Ti_2_Ni and Ni_3_Ti phases [21]. These phases do not undergo martensitic transformation, and from the point of view of shape memory phenomena, their presence is unfavorable. Therefore, special attention was paid to the NiTi wires’ preparation and the temperature range of heat treatments carried out during surface modification. The first step of the surface modification required keeping the wires at 300 °C. Our previous research showed that after surface treatment for about 30 to 60 min, non-equilibrium precipitates of the Ni_4_Ti_3_ phase are formed. Their presence leads to the multi-stage martensitic transformation, which occurs with the additional R-phase. However, this effect disappears due to the thermal conditions used for the second coating deposition step (Figure 8a).

The second stage of introducing the hydroxyapatite coating requires increasing the heat treatment temperature to 800 °C. This temperature ensures the consolidation of hydroxyapatite and increases its adhesion to the NiTi substrate [15]. As follows from the Ni-Ti phase equilibrium system at 800 °C, the parent phase occurs in a broader range of concentrations and is stable. Annealing the alloy at this temperature caused previously formed precipitates to dissolve, leading again to a single-stage martensitic transformation. The measured thermograms show one thermal peak during cooling as well as heating curves (Figure 8a).

All modified clamps were characterized by a single-stage martensitic transformation occurring in the range from −60 °C to 25 °C. This range is comparable to the range that characterized the clamps in their initial state (Figure 5a). Also, for clamps with a modified surface, the A_f_ temperatures were determined and compared with the temperatures of the human and rabbit bodies (Figure 8b). This range decreased by about 8 degrees. However, the remaining margin of the temperature range meets the conditions for the safe use of the clamps. An essential is also the temperature of the beginning of the martensitic transformation M_s_, which for the tested clamps was about −4 °C for those made of wire with a diameter of 1.2 mm and 1.7 mm. For the remaining clamps, it was −5 °C (Figure 8a). An exceptional case was a clamp made of wire with a diameter of 0.8 mm, in which the M_s_ was lowered to −31 °C. From a practical point of view, this means that for an adverse reaction to be triggered (opening the clamp), a fragment of a living organism equipped with an implant would have to be cooled to temperatures lower than −5 °C, which in practice is impossible.

**Figure 9:**
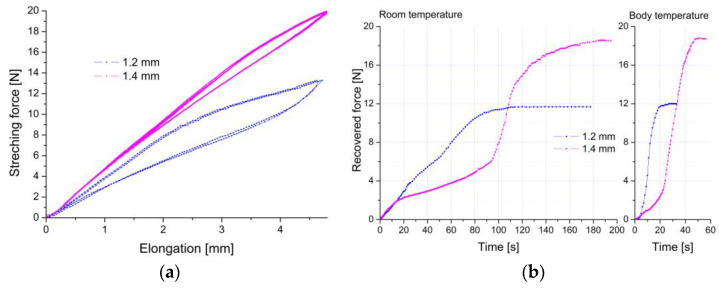
Stretching force versus elongation measured for clamps with modified surfaces (a diameter of 1.2 mm and 1.4 mm) (**a**), and the maximum force leading to the “U” shape (**b**).

Summing up the results obtained so far and looking from a practical point of view, the most advantageous for rabbit applications were wires with a 1.2 or 1.4 mm diameter. The force recovery was again determined using the previously described procedure. For each clamp, two cycles were performed. The measured loops were perfectly overlapped (Figure 9a). This fact proves the repeatability of superelastic behavior and is helpful from a practical point of view. This means that in the case of several attempts to mount a clamp in the drilled hole, it does not lose its properties or characteristics. From the measured data, the maximum recovery force was determined. It was 13.3 N and 19.9 N for the modified 1.2 mm and 1.4 mm clamps, respectively. For comparison with the initial state, these values are plotted in Figure 6b and marked in orange with the “final state” description. Compared to the initial state, it can be concluded that the applied heat treatments did not negatively affect the value of the recovery force needed to form the “U” shape.

Similar to the clamps in the initial state, the force recovery characteristics were determined depending on the elapsed time at both room temperature and the living organism’s temperature. The results are summarized in Figure 9b, while the determined recovery time is compared to those obtained for the clamps in the initial state in Figure 7b. These data are marked with open circles and dashed lines with the description “final state”. The comparison of the results obtained for measurements carried out at room temperature shows that the recovery time for the modified 1.2 mm clamp increased from 96 to 112 s, and for the 1.4 mm clamp, from 150 to 185 s. The results of measurements carried out in conditions of the temperature of a living organism shortened the time needed for full shape recovery. Compared to the clamps in the initial state, this time was slightly longer, and for the modified 1.2 mm clamp, it increased by only 2 s, while for the 1.4 mm one, it increased by 10 s. As previously mentioned, extending the full recovery time had a positive practical effect. The surgeon will have more time to adjust the clamp to the previously drilled holes with these additional seconds.

Summing up all the results obtained for clamps with a modified surface and considering the anatomical conditions of the treatment object, it was decided to use a clamp with a modified surface made of wire with a diameter of 1.2 mm. Therefore, a series of clamps with a 20 mm to 25 mm span and 6–8 mm arms were prepared for the implantation.

### 3.5. Clamp Implantation and Fracture Healing

Following the aim of the study, clamps were implanted in the tibia of both hind limbs. In the case of the left limb, the bone fragments were intentionally fractured and connected using two clamps and stabilizing nails. In order to boost the potentially unwanted effect of the modified clamps on the living organism, the right limb was also equipped with two clamps. Double clamps on each limb were supposed to intensify the possible effect of nickel on the tissues adjacent to the implant. The one-way shape memory effect was predominant in shape recovery.

Then, the implanted clamp was formed in liquid nitrogen into a programmable “U” shape (Figure 1b). The last step was inserting the clamp into the drilled holes. An identical procedure was performed for the tibia of the right limb. In this case, the clamps were applied to the original bone without prior fragmentation. Under the influence of the heat of the rabbit’s body, the clamps returned to their original shape (Figure 1a), pressing the broken bone fragments and clamping the unbroken bone fragments.

**Figure 10:**
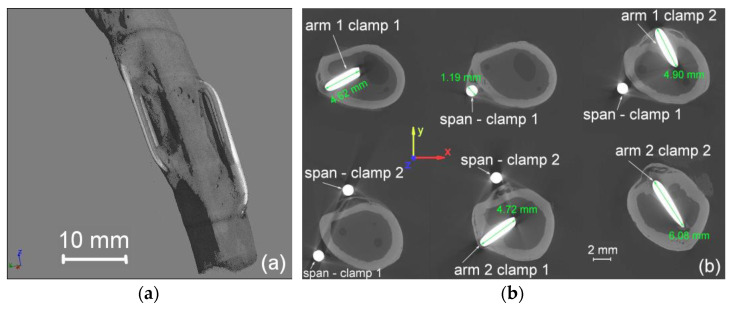
The CT three-dimensional model of the spatial arrangement of clamps in the tibia (**a**) and sample images from the microtomograph showing fragments of implanted clamps (**b**).

After twelve weeks of implantation, the rabbits were euthanized, and bone fragments were collected for testing. First, the clamps’ work areas were checked using microtomography images. Figure 10a shows an exemplary three-dimensional model obtained from microtomography images, showing the relative spatial arrangement of the implanted clamps. They were intentionally placed in an unoverlapping way, while any potential fracture was at a distance of not less than 10 mm from both arms. The next step in the study was checking the position of the arms and spans of the clamps inside the bone. Example images of bone cross-sections with visible arms and spans of both staples spatially located in the tibia are shown in Figure 10b.

Having a series of clamps at his disposal, the surgeon could choose and adjust the size of the clamps appropriately to the semi-minor axis of the bone’s elliptical cross-section, paying attention to not exceeding the assumed minimum penetration depth. Thus, their final length was 6 mm. The cross-section shows that the maximum depth of bone marrow penetration was about 5 mm (Figure 10b—arms 1 and 2 of clamp 1). Clamp 2 was mounted on the semi-major axis with arms 8 mm long. Also, in this case, microtomographic images show that the maximum penetration depth of bone marrow penetration was about 6 mm (Figure 10b—arms 1 and 2 of clamp 2). In all the used and tested cases, no damage, cracking, or destruction of the bone cortex caused by the force of the clamp was found. In addition, microtomographic images confirm the correctness of the procedures.

**Figure 11:**
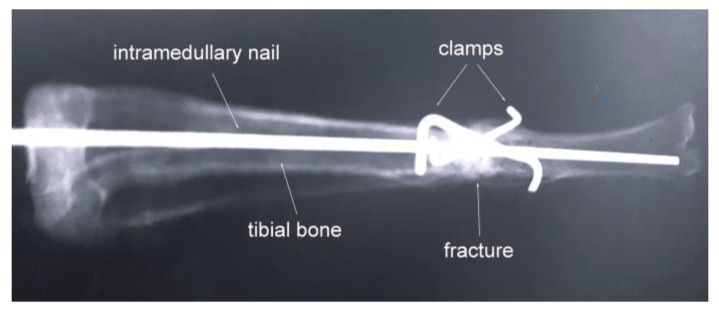
An example of a radiograph taken for a broken tibia with mounted clamps and a nail [22].

In the case of a broken bone, the fragments were first placed in anatomical positions, and holes for mounting the clamps were drilled. Then, the clamps were mounted following the previously described method. In the case of the rabbit, as in humans, the bone fragments undergo osseointegration after about six weeks with standard treatment. After that, X-ray examinations were performed for all cases. An example of a radiographic result is shown in Figure 11.

The analysis of all examined cases shows the formation of a proper joint from bone fragments. The healing process was going correctly. The normal bone junction at the osteotomy site was found radiologically in all rabbits. There were no pathological changes in the tissues surrounding the implants or the regional lymph nodes. Compared to the control group individuals, there were no visible changes in the soft tissues or disturbances in the general condition in the clinical examination.

### 3.6. Histological and SEM-EDS Analysis

**Figure 12:**
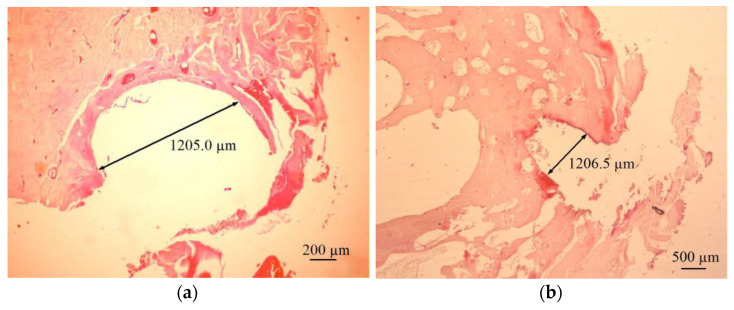
Microscopic images of (**a**) cross- and (**b**) longitudinal sections of the bone from the area of the implanted clamp.

Several representative locations in direct contact with the implanted clamps were selected for histological examination. The samples were cut in both the bone’s cross- and longitudinal sections. The cross-sectional image in which the clamp’s span was located is shown in Figure 12a. In contrast, the cross-section of the longitudinal end of the arm is shown in Figure 12b. At the point of direct contact between the clamp’s surface and the tissue, fragments of the periosteum were characterized by correct structure. Also, the bone tissue revealed the correct structure. Its fragments, directly connected to the clamp’s surface, were supplied with blood. These facts proved the lack of negative impact of clamp modification with the titanium oxy-nitride and hydroxyapatite layers on the biological environment.

**Figure 13:**
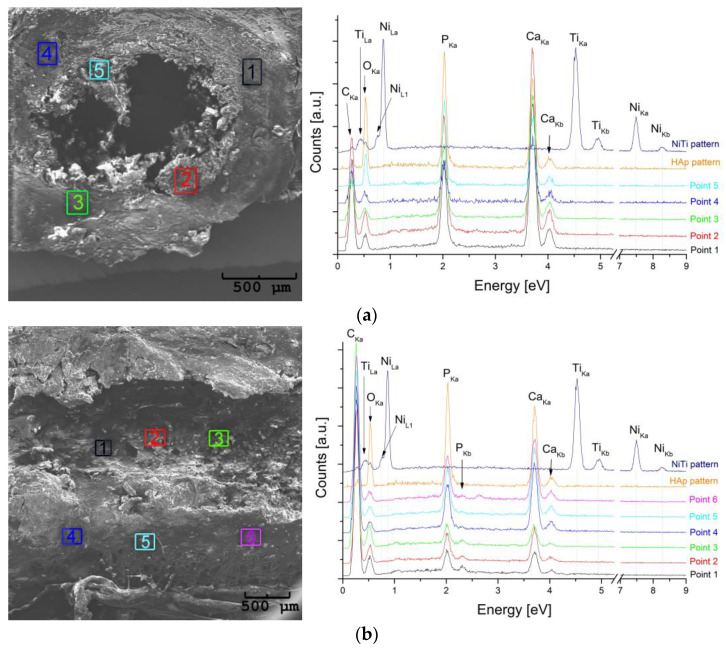
SEM images of (**a**) cross- and (**b**) longitudinal sections of the bone from the area of the implanted clamp with the EDS spectra measured at related points.

In order to confirm the assumed protective effect of the coating/layer, microscopic observation was carried out with the chemical composition measurement. The elements were detected in the bone tissues—in cross sections (Figure 13a—points 1 to 5) and longitudinal sections (Figure 13b—points 4–6). In addition, the tests were carried out on the walls of the canal remaining after the extraction of the clamp (Figure 13b—points 1–3). The places dedicated to determining the chemical composition were rectangular with approximate dimensions of 200 μm × 500 μm and marked with colors corresponding to the colors of the measured spectra. Also, measurements were performed for the NiTi wire and the hydroxyapatite used for surface modification. The received spectra were marked in blue (NiTi) and orange (HAp).

The comparison of the spectra shows that the spectral lines related to nickel and titanium did not appear in the spectra measured for the bone. Calcium, phosphorus, and oxygen (as the components of hydroxyapatite—a bone-building material) were stated in all studied areas. Noteworthy is the fact that the penetration depth of the electron beam is about a few micrometers. Hence, these results confirm that the tested bone volumes are free of nickel and titanium. Consequently, the protective nature of the layer/coating applied to the clamps was confirmed.

## 4. Conclusions

The results of the conducted research can be summarized by drawing the following conclusions:

- From the clamps made of NiTi wire (nickel content 50.6–50.7 at%) with diameters from 0.8 to 1.7 mm with the one-way shape memory effect, it is possible to receive force recovery of 6 to 80 N—according to the range of elastic deformations of a rabbit’s hind bones.
- The introduction of a two-stage modification of the surface of NiTi clamps, consisting of the formation of a titanium oxy-nitride layer and a hydroxyapatite coating, does not limit the temperature range of applicability of the one-way shape memory effect in the case of living organisms with a body temperature of 36.6 °C to 38 °C.
- The time of the clamp’s (with a modified surface) return to the initially programmed shape caused by the temperature of the living body, and obtaining the full recovery force was 24 s and 48 s for the clamp with a modified surface made of wire with a diameter of 1.2 mm and 1.4 mm, respectively.
- Compared to the control group, the results of histological tests carried out for the implantation group confirm the lack of harmful effects from the interaction of the NiTi alloy with a living organism. This proved the protective effect of the layers/coatings applied to the NiTi clamp. Therefore, clamps with a modified surface can be successfully used in bone surgery on small and medium-sized animals with an extended duration of operation of up to 12 weeks.

## Figures and Tables

**Table 1:** Average chemical composition (at.%) determined at wires’ cross-section.

Wire Diameter [mm]	Ni [at%]	Ti [at.%]
0.8	50.6 ± 0.4	49.4 ± 0.3
1.0	50.5 ± 0.4	49.5 ± 0.3
1.2	50.7 ± 0.4	49.3 ± 0.3
1.4	50.6 ± 0.4	49.4 ± 0.3
1.7	50.4 ± 0.4	49.6 ± 0.3

## Data Availability

The data presented in this study are available on request from the corresponding authors.
